# Diagnostic performance of controlled attenuation parameter for grading hepatic steatosis in MASLD: an MRI-PDFF-referenced study in a Chinese cohort

**DOI:** 10.3389/fmed.2026.1836752

**Published:** 2026-05-19

**Authors:** Siying Wang, Dongfang Shang, Chenlu Zhao, Lihui Zhang, Sutong Liu, Qing Zhao, Qiang Zhang, Wenxia Zhao

**Affiliations:** Department of Gastroenterology and Hepatology, The First Affiliated Hospital of Henan University of Chinese Medicine, Zhengzhou, China

**Keywords:** controlled attenuation parameter, diagnostic accuracy, hepatic steatosis grading, MRI-PDFF, MASLD

## Abstract

**Objective:**

This study aimed to evaluate the diagnostic accuracy of the controlled attenuation parameter (CAP) for grading hepatic steatosis in metabolic dysfunction-associated steatotic liver disease (MASLD), using magnetic resonance imaging-derived proton density fat fraction (MRI-PDFF) as the non-invasive reference standard.

**Methods:**

This single-center retrospective study included 120 participants (17 healthy controls and 103 patients with MASLD) who underwent MRI-PDFF, transient elastography (TE), and laboratory testing within predefined time intervals. Participants were stratified by MRI-PDFF values into no (S0, *n* = 17), mild (S1, *n* = 20), moderate (S2, *n* = 59), or severe (S3, *n* = 24) steatosis. Group comparisons were performed using ANOVA or Kruskal-Wallis tests, correlations were assessed with Pearson or Spearman coefficients, and independent factors associated with CAP were identified by multivariate linear regression. The diagnostic performance of CAP was evaluated using receiver operating characteristic (ROC) curve analysis. Bootstrap resampling (2000 iterations) was used to estimate the 95% confidence intervals (CIs) for the optimal CAP cut-off values for each steatosis grade.

**Results:**

CAP values increased progressively with steatosis severity (S3 > S2 > S1 > S0; all *p* < 0.001). Hepatic steatosis grade was independently associated with CAP values after adjusting for confounders (*p* < 0.001). CAP showed good diagnostic performance for identifying ≥S1 (area under the curve [AUC] = 0.924) and ≥S2 steatosis (AUC = 0.947), and acceptable performance for identifying S3 steatosis (AUC = 0.837). The optimal CAP cut-offs were 239 dB/m (95% CI: 235.5–240.0) for ≥S1, 278 dB/m (95% CI: 273.0–307.5) for ≥S2, and 314 dB/m (95% CI: 303.5–357.5) for S3, with corresponding sensitivities of 95.0, 94.9, and 91.7%, and specificities of 88.2, 83.8, and 62.5%.

**Conclusion:**

CAP showed good diagnostic performance for grading hepatic steatosis in MASLD and may serve as a practical non-invasive tool for steatosis assessment, particularly in resource-limited settings where MRI-PDFF is not readily available.

## 1 Introduction

Metabolic dysfunction-associated steatotic liver disease (MASLD), formerly encompassed within the spectrum of non-alcoholic fatty liver disease, refers to hepatic steatosis occurring in the context of cardiometabolic risk and is strongly associated with obesity, type 2 diabetes mellitus (T2DM), and other metabolic abnormalities (1, 2). As disease severity increases, the risks of liver fibrosis, cirrhosis-related complications, and hepatocellular carcinoma (HCC) also increase. Therefore, accurate risk stratification and tailored management of MASLD are of major clinical importance (3, 4). Although liver biopsy remains the reference standard for grading steatosis, its utility is constrained by invasiveness, limited reproducibility, and sampling variability, underscoring the demand for robust non-invasive diagnostic alternatives (5, 6).

Current non-invasive modalities mainly comprise serum-based biomarkers and imaging techniques. Serum models—such as the Fatty Liver Index (FLI), Hepatic Steatosis Index (HSI), and SteatoTest—are inexpensive and straightforward to implement (7). However, their diagnostic performance may be affected by coexisting metabolic abnormalities, and the lack of standardized cut-off values limits their clinical utility. Therefore, these tools should not be used as standalone diagnostic measures for individual patient management (8). Conventional ultrasound is widely employed for steatosis detection; however, it suffers from limited sensitivity in mild steatosis and does not provide quantitative fat assessment (9–11). Computed tomography (CT) can diagnose hepatic steatosis by comparing hepatic and splenic attenuation values (12, 13). Yet, due to substantial radiation exposure and relatively high cost, CT is generally unsuitable for routine screening (14, 15).

The advent of magnetic resonance imaging-derived proton density fat fraction (MRI-PDFF) has provided a non-invasive and accurate methodology for quantifying hepatic steatosis (16). This technique facilitates fat quantification across the entire liver or within specific regions of interest, delivering high accuracy, excellent reproducibility, and whole-liver coverage (17). Furthermore, MRI-PDFF exhibits high sensitivity for detecting mild steatosis and correlates strongly with histologic assessments (18, 19). As a result, it is increasingly regarded in numerous studies as a non-invasive reference standard that serves as an alternative to liver biopsy (20–22). However, its high cost and limited availability restrict its widespread use as a first-line screening modality (23).

In contrast, the controlled attenuation parameter (CAP), integrated into vibration-controlled transient elastography (VCTE), offers several practical advantages, including non-invasiveness, rapid acquisition, ease of use, and affordability. It also allows concurrent liver stiffness measurement (LSM), highlighting its promise for broad clinical implementation (24–26). CAP has been incorporated into or discussed in several hepatology practice guidelines as a useful non-invasive tool for steatosis assessment (27–29). Multiple studies have reported significant correlations between CAP values and histologic steatosis grades (30–33). A meta-analysis of 10,537 individuals (34) revealed that CAP delivers robust diagnostic performance across steatosis stages, with area under the receiver operating characteristic curve (AUROC) values of 0.924, 0.794, and 0.778 for S1, S2, and S3, respectively. In a cross-sectional and longitudinal investigation, An et al. (35) comprehensively compared CAP and MRI-PDFF for tracking liver fat dynamics, demonstrating significant correlation between the two modalities. These findings support the complementary use of CAP and MRI-PDFF in the non-invasive assessment of hepatic steatosis, particularly in Asian populations.

Several challenges remain in optimizing the diagnostic utility of CAP. Firstly, most established CAP cut-offs are derived from Western cohorts, and their validity in Chinese MASLD patients has not been adequately assessed (36). Secondly, CAP measurements can be influenced by factors such as obesity and restricted intercostal spaces, and consensus regarding optimal diagnostic thresholds is still lacking (37). These issues currently impede the precise and standardized application of CAP in clinical settings.

Using MRI-PDFF as a non-invasive reference standard, this study aimed to evaluate the diagnostic performance of CAP for grading hepatic steatosis, derive population-specific CAP cut-off values for a Chinese cohort, and identify factors associated with CAP values.

## 2 Materials and methods

### 2.1 Study participants and design

**Figure 1:**
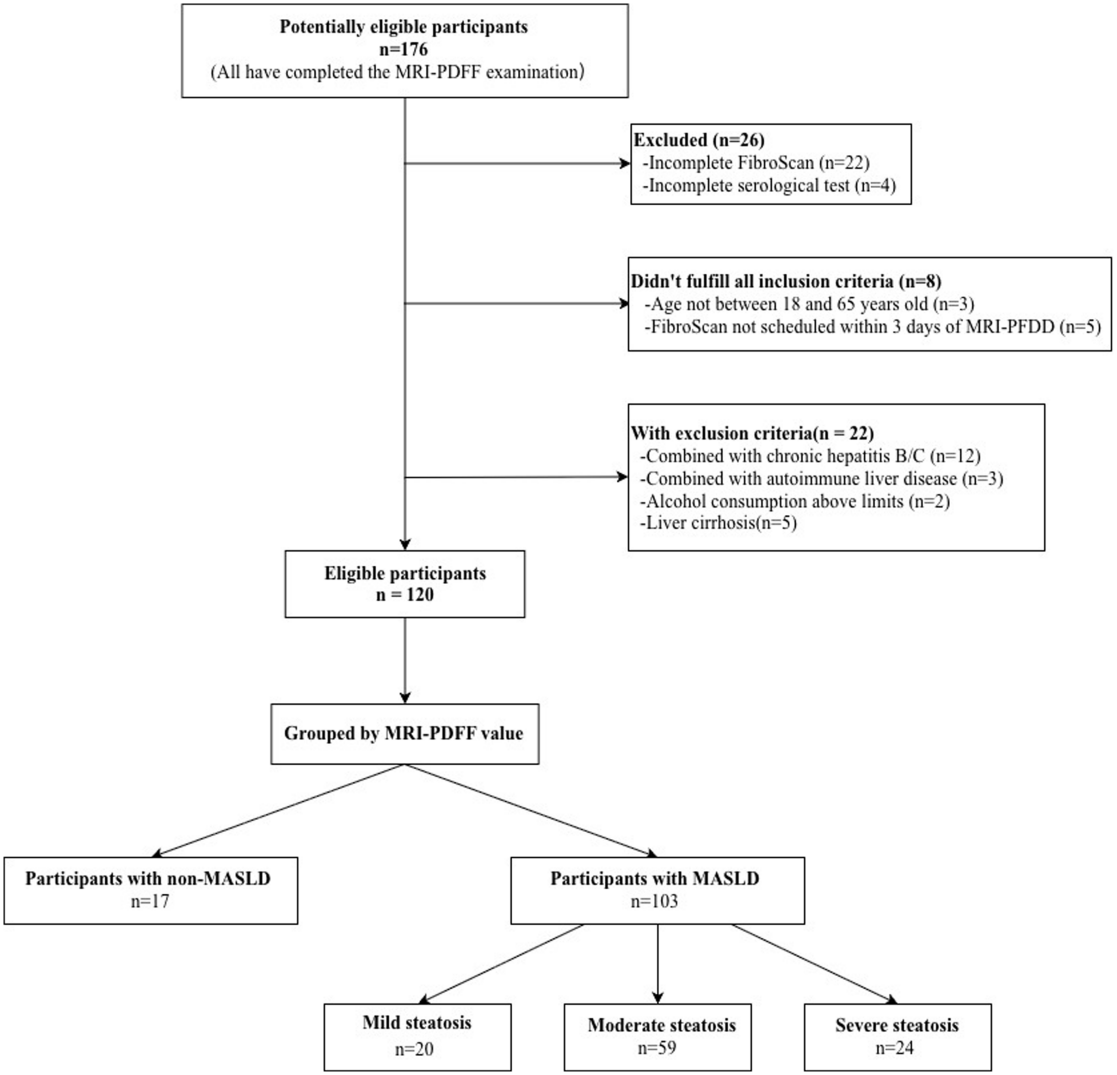
Participant enrollment flow diagram. This flowchart delineates the participant screening process. Initially, 176 individuals were assessed for eligibility. Following application of the inclusion/exclusion criteria, 120 participants constituted the final study cohort, which included 103 patients with MASLD and 17 healthy controls. MASLD, metabolic dysfunction-associated steatotic liver disease; CAP, controlled attenuation parameter; MRI-PDFF, magnetic resonance imaging-derived proton density fat fraction.

This retrospective study included individuals who underwent MRI-PDFF, transient elastography (TE), and laboratory investigations at the First Affiliated Hospital of Henan University of Chinese Medicine between March 2024 and March 2025 and met the predefined inclusion and exclusion criteria. The final cohort consisted of 120 participants, including 17 healthy controls and 103 patients classified according to the *Guidelines for the Prevention and Treatment of Metabolic-Associated Fatty Liver Disease (2024 Edition)* (38); given its substantial overlap with the contemporary MASLD framework, the term MASLD is used throughout this manuscript for consistency with current international nomenclature. A participant flow diagram is shown in Figure 1.

Demographic and anthropometric data, including age, sex, height, body weight (BW), and waist circumference (WC), were collected from the medical records. Body mass index (BMI) was calculated as weight (kg) divided by height squared (m^2^).

#### 2.1.1 Inclusion criteria

- Age between 18 and 65 years.
- Completion of all required examinations within specified timeframes: MRI-PDFF, transient elastography (with CAP measurement), and laboratory tests, with a maximum interval of 3 days between MRI-PDFF and TE, and 1 week for laboratory tests.

#### 2.1.2 Exclusion criteria

- Presence of other chronic liver diseases, including viral hepatitis (such as Hepatitis A, Hepatitis B, and Hepatitis C) infection, drug-induced liver injury, autoimmune hepatitis, or Wilson’s disease.
- Excessive alcohol consumption (defined as ≥210 g/week for men or ≥140 g/week for women).
- Presence of implantable pacemakers, significant ascites, unhealed wounds in the right upper quadrant, or pregnancy/lactation.
- Diagnosis of liver cirrhosis or hepatocellular carcinoma.
- Use of immunosuppressive therapy within the past year.

### 2.2 Controlled attenuation parameter (CAP) measurement

**Figure 2:**
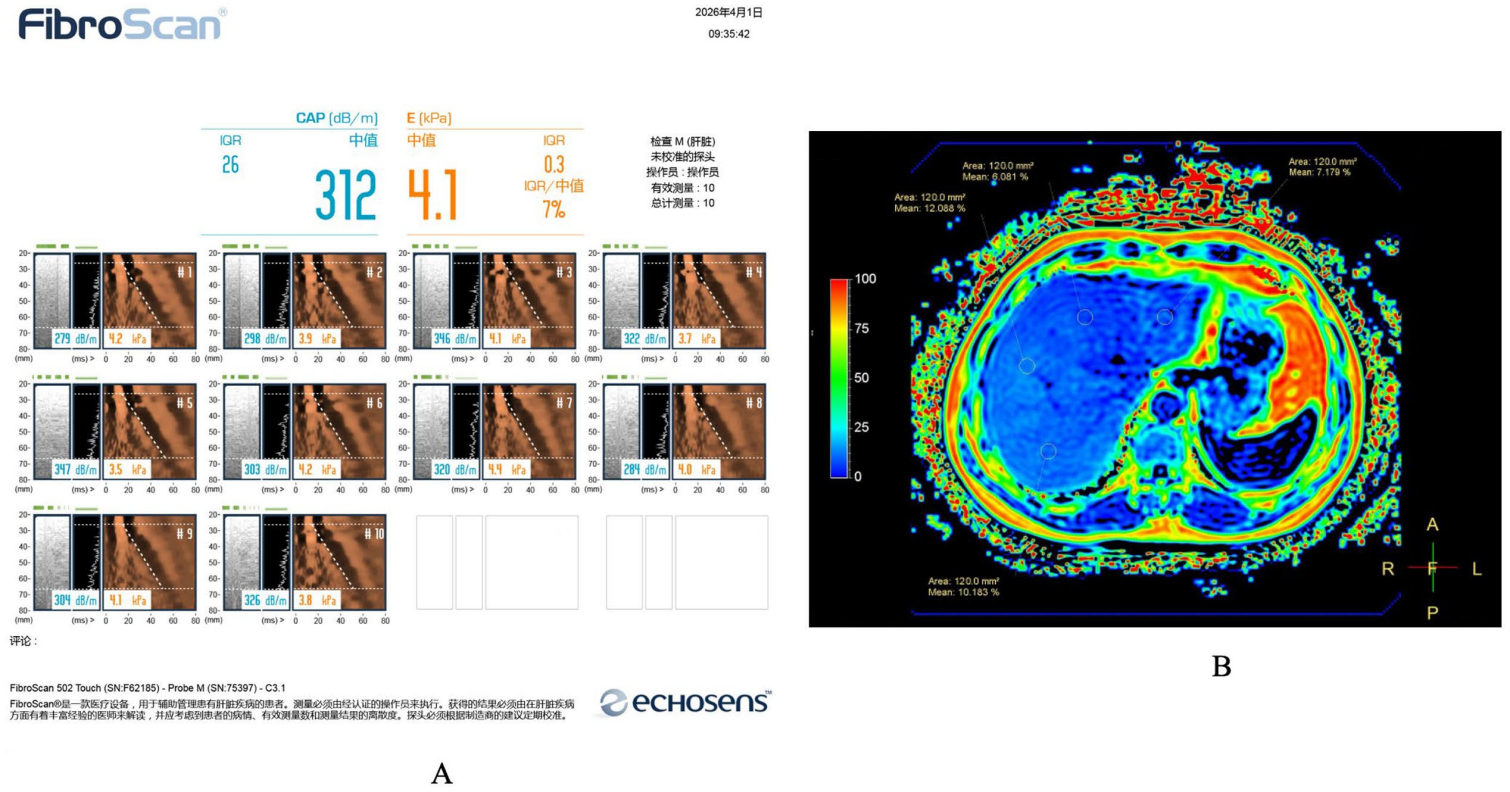
Representative FibroScan/CAP and MRI-PDFF images used for hepatic steatosis assessment. **(A)** Representative FibroScan/VCTE report showing simultaneous acquisition of controlled attenuation parameter (CAP) and liver stiffness measurement (LSM) using the M probe. The report also displays quality-control parameters and repeated valid measurements obtained during the examination. **(B)** Representative axial MRI-PDFF image acquired at the mid-liver level, showing four circular regions of interest (ROIs) (each approximately 120 mm^2^) placed in the right anterior, right posterior, left medial, and left lateral hepatic segments. Major vessels, bile ducts, focal lesions, and imaging artifacts were avoided. The mean PDFF value derived from the four ROIs was used for analysis. MRI-PDFF, magnetic resonance imaging-derived proton density fat fraction; CAP, controlled attenuation parameter; VCTE, vibration-controlled transient elastography; LSM, liver stiffness measurement; ROI, region of interest.

CAP measurements were acquired using a FibroScan® 502 Touch device (Echosens, France) equipped with an M probe. The skin-to-liver capsule distance (SLCD) was not routinely measured. Although guidelines recommend the XL probe for patients with a body mass index (BMI) ≥ 30 kg/m^2^ or SLCD >25 mm (39, 40), the XL probe was not widely available in China during the study period. Therefore, the M probe was used for all participants, consistent with local clinical practice and previous studies (31, 41). Standard quality-control criteria were applied to ensure measurement reliability. All examinations were conducted by one experienced technician who was certified in the operation of the device and followed a standardized protocol. Participants were positioned supine with the right arm fully abducted to maximize the intercostal window. Measurements were obtained from the 7th to 9th intercostal spaces along the right mid-axillary to anterior axillary line, ensuring the probe remained perpendicular to the skin surface with appropriate pressure. A valid examination required at least 10 acquisitions, with a success rate of ≥60% and an interquartile range (IQR) to median ratio of less than 30%. Liver stiffness measurement (LSM) was recorded concurrently. A representative FibroScan/CAP report is shown in Figure 2A.

### 2.3 Laboratory assessments

Serum biochemical parameters were quantified using a Beckman Coulter AU5811 automated biochemical analyzer. The analyzed parameters included platelet count (PLT), albumin (Alb), total bilirubin (Tbil), alanine aminotransferase (ALT), aspartate aminotransferase (AST), gamma-glutamyl transferase (GGT), alkaline phosphatase (ALP), fasting blood glucose (GLU), low-density lipoprotein cholesterol (LDL-C), high-density lipoprotein cholesterol (HDL-C), triglycerides (TG), total cholesterol (TC), free fatty acids (NEFA), and fasting insulin. Insulin resistance was additionally estimated using HOMA-IR (homeostatic model assessment of insulin resistance), calculated as fasting insulin (μIU/mL) × fasting glucose (mmol/L)/22.5.

### 2.4 Magnetic resonance imaging-derived proton density fat fraction (MRI-PDFF) acquisition

MRI-PDFF was acquired on a Philips Prodiva 3.0 T scanner utilizing the IDEAL-IQ sequence for fat quantification. The acquisition parameters were as follows: repetition time (TR) 10 ms, echo time (TE) 4 ms, slice thickness 3 mm, and interslice gap 20 mm. Four circular regions of interest (ROIs), each approximately 120 mm^2^, were placed in the medial and lateral segments of the left lobe and the anterior and posterior segments of the right lobe, while avoiding major bile ducts, blood vessels, focal lesions, and imaging artifacts (42). The mean PDFF value derived from these four ROIs was calculated and used for subsequent analysis. A representative MRI-PDFF image showing ROI placement is presented in Figure 2B. Hepatic steatosis was graded according to established MRI-PDFF thresholds: S0 (normal, <5%), S1 (mild, ≥5 to <10%), S2 (moderate, ≥10 to <25%), and S3 (severe, ≥25%) (16, 43).

### 2.5 Calculation of non-invasive metabolic indices and combined analysis

To further explore whether combining CAP with simple non-invasive metabolic indices could improve diagnostic performance for hepatic steatosis, the fatty liver index (FLI), visceral adiposity index (VAI), and triglyceride-glucose (TyG) index were additionally calculated. FLI was calculated as follows:

FLI = [*e*^(0.953 × ln TG + 0.139 × BMI + 0.718 × ln GGT + 0.053 × WC − 15.745)^/(1 + *e*^(0.953 × ln TG + 0.139 × BMI + 0.718 × ln GGT + 0.053 × WC − 15.745)^)] × 100, where TG was expressed in mmol/L, BMI in kg/m^2^, GGT in U/L, and WC in cm.

VAI was calculated using sex-specific formulas: for males, VAI = [WC/(39.68 + 1.88 × BMI)] × (TG/1.03) × (1.31/HDL-C); for females, VAI = [WC/(36.58 + 1.89 × BMI)] × (TG/0.81) × (1.52/HDL-C), where WC was expressed in cm, BMI in kg/m^2^, and TG and HDL-C in mmol/L.

The TyG index was calculated as ln [fasting TG (mg/dL) × fasting glucose (mg/dL)/2].

To evaluate the additional value of combining CAP with these metabolic indices, binary logistic regression models were constructed separately by combining CAP with FLI, VAI, or TyG for identifying ≥S1, ≥S2, and S3 steatosis. The predicted probabilities derived from these combined models were then used for ROC analysis.

### 2.6 Ethical considerations

The study protocol received approval from the Ethics Committee of The First Affiliated Hospital of Henan University of Chinese Medicine (Approval Number: 2025HL-330-01). The requirement for informed consent was waived due to the retrospective nature of the study.

### 2.7 Statistical analysis

All statistical analyses were performed using SPSS (version 26.0), GraphPad Prism (version 10.0), and R (version 4.5.1) software. Continuous variables with a normal distribution are presented as mean ± standard deviation (SD). Non-normally distributed data are summarized as median with interquartile range (IQR). Categorical variables are reported as numbers and percentages.

Comparisons between two groups were conducted using the *t*-test. For comparisons among more than two groups, one-way analysis of variance (ANOVA) was employed, followed by the least significant difference (LSD) *post-hoc* test for pairwise comparisons. Group comparisons for non-normally distributed data were performed using the Kruskal-Wallis test, with Bonferroni adjustment for multiple comparisons. Comparisons of categorical variables were made using the Chi-square (*χ*^2^) test.

Pearson’s correlation coefficient was used to assess the relationship between two normally distributed continuous variables. Spearman’s rank correlation coefficient was used for non-normally distributed data or ordinal variables. Variables showing significant associations in the correlation analysis (*p* < 0.05) and considered clinically relevant were included in univariate linear regression models. Variables with *p* < 0.05 in the univariate analysis were subsequently entered into the multivariable linear regression model. Variables that remained significant (*p* < 0.05) in the final multivariate model were identified as independent factors associated with CAP values. Multicollinearity was assessed using the variance inflation factor (VIF), with a VIF > 10 indicating severe multicollinearity. The Durbin-Watson (D-W) statistic was used to evaluate residual autocorrelation, with a value near 2 suggesting its absence.

Using MRI-PDFF as the reference standard, receiver operating characteristic (ROC) curves were generated to evaluate the diagnostic performance of CAP for different grades of hepatic steatosis in MASLD. The area under the ROC curve (AUC) was calculated to assess overall accuracy, while sensitivity and specificity were determined at optimal cut-off points. The optimal cut-off value for diagnosing each grade of hepatic steatosis was selected by maximizing Youden’s *J* index. Bootstrap resampling with 2000 iterations was used to estimate the 95% confidence intervals (CIs) for the optimal cut-off values for each steatosis grade. A two-sided *p*-value of less than 0.05 was considered statistically significant.

Additional ROC analyses were performed to assess the diagnostic performance of FLI, VAI, and TyG individually and in combination with CAP for identifying ≥S1, ≥S2, and S3 steatosis. The area under the ROC curve (AUC), sensitivity, specificity, and Youden index were calculated for each model. Differences in AUC between CAP alone, the individual metabolic indices, and the combined models were compared using DeLong’s test.

## 3 Results

### 3.1 Baseline clinical characteristics

**Figure 3:**
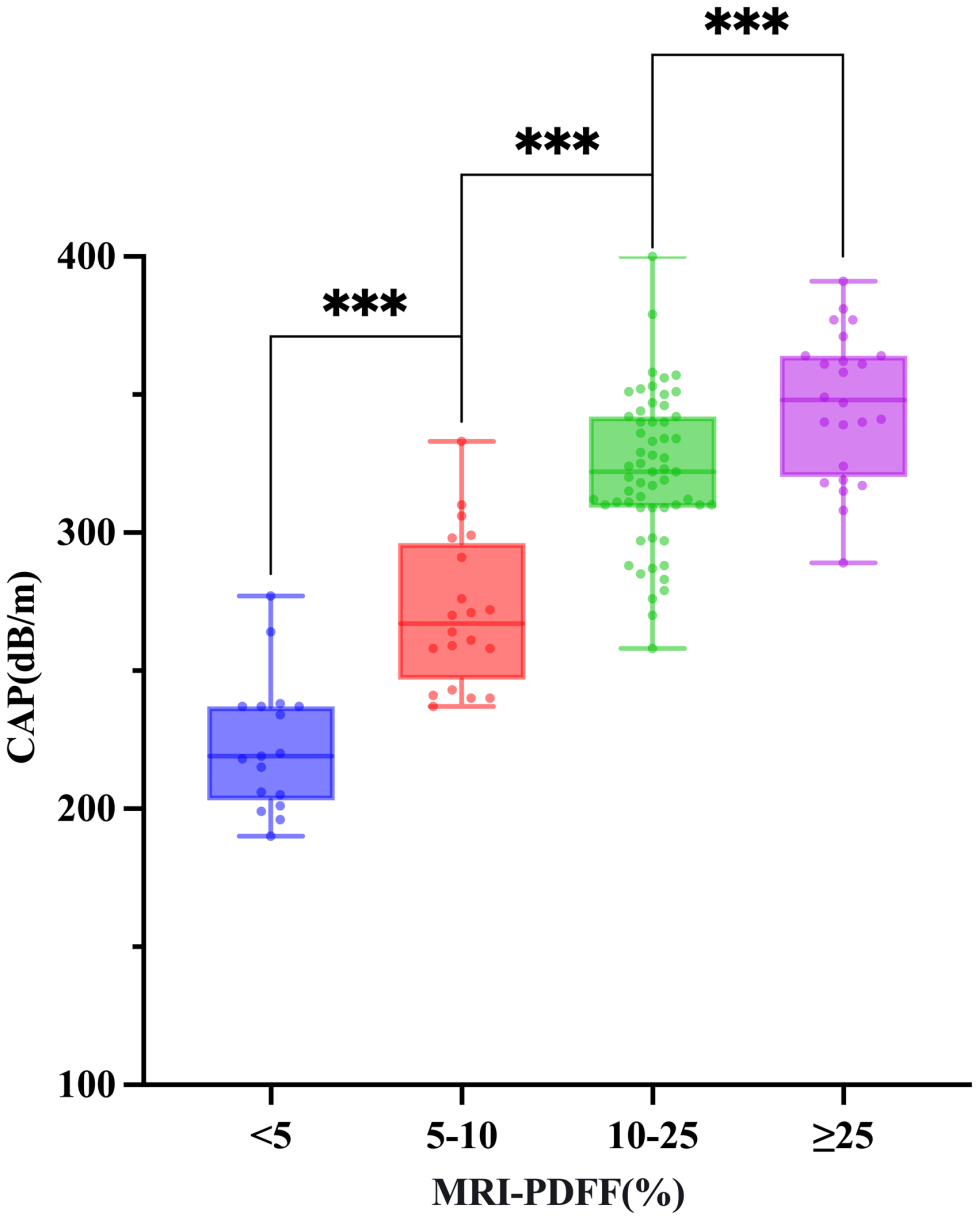
Distribution of CAP values according to hepatic steatosis grade. Box plot showing the progressive increase in CAP values across MRI-PDFF-defined steatosis grades (S0 to S3). Statistical significance was assessed using one-way ANOVA with post-hoc testing (***p < 0.001). CAP, controlled attenuation parameter; MRI-PDFF, magnetic resonance imaging-derived proton density fat fraction.

The final analysis included 120 participants, consisting of 89 males (74.2%) and 31 females (25.8%). The mean age was 36.40 ± 9.57 years, and the median BMI was 27.28 kg/m^2^ (interquartile range [IQR]: 4.07). Based on MRI-PDFF values, participants were stratified into four steatosis grades: S0 (none, *n* = 17), S1 (mild, *n* = 20), S2 (moderate, *n* = 59), and S3 (severe, *n* = 24). Significant intergroup differences were observed for WC, BW, BMI, PLT, ALT, AST, GGT, TC, TG, LDL-C, CAP, and LSM (all *p* < 0.05). Post-hoc pairwise comparisons revealed a progressive increase in CAP values across steatosis grades (S3 > S2 > S1 > S0; all *p* < 0.001) (Figure 3). Pairwise comparisons showed that several anthropometric and liver-related parameters, including WC, BW, BMI, PLT, AST, ALT, and LSM, were significantly higher in the more advanced steatosis groups than in the S0 group (all *p* < 0.05). In contrast, no significant differences were found among groups for sex, age, height, Tbil, Alb, ALP, GLU, HDL-C, NEFA, fasting insulin or HOMA-IR (all *p* > 0.05) (Table 1).

**Table 1:** Comparison of clinical characteristics across MASLD steatosis grades.

Characteristic	S0 (*n* = 17)	S1 (*n* = 20)	S2 (*n* = 59)	S3 (*n* = 24)	*X^2^*/*F*/*Z*	*p*-value
Male, *n* (%)	12 (70.60)	15 (75.00)	45 (76.30)	17 (70.80)	0.392	0.942
Age (years)	39.00 ± 12.08	35.50 ± 8.42	37.46 ± 9.27	32.71 ± 8.62	1.955	0.125
WC (cm)	76.80 (11.30)	88.40 (12.98)	93.40 (12.80)^a^	92.71 (25.36)^a^	26.436	<0.001
Height (m)	1.70 (0.08)	1.74 (0.13)	1.71 (0.08)	1.72 (0.10)	2.020	0.568
BW (kg)	71.61 ± 6.85	79.12 ± 10.27	83.46 ± 13.50^a^	83.96 ± 14.60^a^	4.528	0.005
BMI (kg/m^2^)	24.20 (3.20)	26.74 (2.38)	28.42 (4.09)^a^	27.95 (4.34)^a^	26.884	<0.001
PLT (10^9^/L)	222.76 ± 52.32	234.45 ± 43.22	255.63 ± 46.38^a^	253.13 ± 49.87^a^	2.727	0.047
Tbil (μmol/L)	18.90 (6.30)	14.50 (9.40)	14.60 (7.20)	14.45 (8.80)	4.309	0.230
Alb (g/L)	46.86 ± 4.12	46.01 ± 3.66	46.51 ± 3.44	47.15 ± 3.08	0.432	0.730
ALT (U/L)	32.60 (22.90)	45.90 (34.00)	62.30 (42.90)^a^	66.30 (26.90)^ab^	28.940	<0.001
AST (U/L)	25.90 (12.20)	30.95 (10.60)	37.60 (18.70)^a^	36.95 (18.20)^a^	18.641	<0.001
ALP (U/L)	73.50 (30.40)	91.30 (32.60)	83.80 (21.20)	79.40 (27.90)	6.079	0.108
GGT (U/L)	34.40 (25.65)	52.05 (68.22)	46.00 (25.00)	55.80 (39.00)^a^	9.315	0.025
GLU (mmol/L)	5.34 (0.68)	5.43 (0.75)	5.31 (0.69)	5.21 (0.59)	3.121	0.373
TC (mmol/L)	4.58 (0.83)	5.08 (0.86)	5.49 (1.24)^a^	4.95 (1.20)	18.445	<0.001
TG (mmol/L)	1.19 (0.82)	1.29 (0.85)	2.05 (1.27)^ab^	1.59 (1.42)	16.377	0.001
HDL-C (mmol/L)	1.12 (0.35)	1.21 (0.27)	1.13 (0.29)	1.12 (0.22)	3.588	0.310
LDL-C (mmol/L)	2.82 ± 0.72	3.21 ± 0.64	3.61 ± 0.69^ab^	3.22 ± 0.59^c^	7.098	<0.001
NEFA (mmol/L)	0.56 ± 0.11	0.62 ± 0.17	0.55 ± 0.19	0.63 ± 0.21	1.608	0.191
Insulin (μIU/ml)	16.10 (8.00)	16.30 (7.10)	17.20 (10.50)	15.50 (8.80)	2.898	0.408
HOMA-IR	3.64 ± 1.56	3.87 ± 1.52	4.48 ± 1.73	4.09 ± 1.95	1.383	0.252
CAP (dB/m)	223.12 ± 23.90	271.35 ± 27.07^a^	322.14 ± 26.99^ab^	346.38 ± 26.33^abc^	92.808	<0.001
LSM (kPa)	4.60 (2.30)	5.50 (2.20)	5.90 (3.50)^a^	6.n (3.10)^a^	15.926	0.001

Post-hoc pairwise comparisons were performed. Different superscript letters (a, b, c) denote significant differences (*p* < 0.05) between groups: a vs. S0; b vs. S1; c vs. S2. Data are presented as mean ± standard deviation, median (interquartile range), or number (percentage). MASLD, metabolic dysfunction-associated steatotic liver disease; WC, waist circumference; BMI, body mass index; PLT, platelet count; Tbil, total bilirubin; Alb, albumin; ALT, alanine aminotransferase; AST, aspartate aminotransferase; ALP, alkaline phosphatase; GGT, gamma-glutamyl transferase; GLU, fasting glucose; TC, total cholesterol; TG, triglycerides; HDL-C, high-density lipoprotein cholesterol; LDL-C, low-density lipoprotein cholesterol; NEFA, free fatty acids; HOMA-IR, homeostatic model assessment of insulin resistance; CAP, controlled attenuation parameter; LSM, liver stiffness measurement; IQR, interquartile range; SD, standard deviation.

### 3.2 Factors associated with CAP values

#### 3.2.1 Correlation analysis

CAP values were positively correlated with WC (*r* = 0.496, *p* < 0.001), BMI (*r* = 0.428, *p* < 0.001), PLT (*r* = 0.245, *p* = 0.007), ALT (*r* = 0.369, *p* < 0.001), AST (*r* = 0.249, *p* = 0.006), GGT (*r* = 0.207, *p* = 0.024), TG (*r* = 0.213, *p* = 0.019), LDL-C (*r* = 0.263, *p* = 0.004), and LSM (*r* = 0.432, *p* < 0.001). CAP values were weakly negatively correlated with age (*r* = −0.222, *p* = 0.015). No significant correlations were observed with sex, Tbil, Alb, ALP, GLU, TC, HDL-C, NEFA, fasting insulin, or HOMA-IR (all *p* > 0.05). In addition, hepatic steatosis grade showed a strong positive correlation with CAP values (*r* = 0.772, *p* < 0.001).

#### 3.2.2 Linear regression analysis

Variables showing significant correlations (*p* < 0.05), including age, WC, BMI, PLT, ALT, AST, GGT, TG, LDL-C, LSM, and hepatic steatosis grade (with S0 as the reference group), were entered into univariate linear regression models. Univariate analysis identified age, WC, BMI, PLT, ALT, TG, LDL-C, LSM, and hepatic steatosis grade as factors significantly associated with CAP values (all *p* < 0.05). Variables with *p* < 0.05 in the univariate analysis were subsequently entered into the multivariable linear regression model. These variables were also considered clinically relevant based on their established associations with metabolic dysfunction and liver disease.

After adjustment for potential confounders (age, WC, BMI, PLT, ALT, TG, LDL-C, and LSM), MRI-PDFF-defined steatosis grade remained independently associated with CAP values (all *p* < 0.001). LSM (*p* = 0.008) and age (*p* = 0.013) also remained independently associated with CAP values, whereas WC, BMI, PLT, ALT, TG, and LDL-C were not retained as independent factors in the final model. All variables had VIF values below 4, indicating no evidence of severe multicollinearity. The Durbin-Watson statistic was 1.892, suggesting no substantial residual autocorrelation (Table 2).

**Table 2:** Factors associated with CAP values in univariate and multivariate linear regression analyses.

Variables	Univariate analysis		Multivariate analysis		
	*B* (95% CI)	*p*-value	*B* (95% CI)	*p*-value	VIF
Age	−1.119 (−2.014, −0.223)	0.015	−0.652 (−1.164, −0.139)	0.013	1.228
WC	2.217 (1.597, 2.838)	<0.001	0.688 (−0.023, 1.399)	0.058	3.631
BMI	6.828 (4.516, 9.139)	<0.001	−0.951 (−3.350, 1.448)	0.434	3.291
PLT	0.244 (0.068, 0.419)	0.007	−0.011 (−0.111, 0.089)	0.827	1.193
ALT	0.356 (0.131, 0.582)	0.002	−0.114 (−0.247, 0.18)	0.089	1.253
AST	−0.025 (−0.248, 0.198)	0.826	—	—	—
GGT	0.118 (−0.061, 0.298)	0.194	—	—	—
TG	9.314 (2.458, 16.169)	0.008	0.913 (−2.911, 4.737)	0.637	1.155
LDL-C	17.621 (5.844, 29.397)	0.004	5.127 (−1.834, 12.089)	0.147	1.282
LSM	7.417 (4.500, 10.333)	<0.001	2.574 (0.693, 4.455)	0.008	1.350
Steatosis grade (ref: S0)					
S1	48.232 (30.941, 65.524)	<0.001	41.408 (24.711, 58.106)	<0.001	2.765
S2	99.018 (84.589, 113.447)	<0.001	86.514 (69.434, 103.594)	<0.001	3.757
S3	123.257 (106.641, 139.874)	<0.001	107.691 (89.377, 126.005)	<0.001	1.995

*The multivariate model was adjusted for age, WC, BMI, PLT, ALT, TG, LDL-C, and LSM. Dashes indicate variables not entered into the multivariable model because they were not significant in univariate analysis. Durbin-Watson statistic = 1.892. WC, waist circumference; BMI, body mass index; PLT, platelet count; ALT, alanine aminotransferase; AST, aspartate aminotransferase; TG, triglycerides; LDL-C, low-density lipoprotein cholesterol; LSM, liver stiffness measurement; VIF, variance inflation factor.

### 3.3 Diagnostic performance of CAP for hepatic steatosis grading

Receiver operating characteristic (ROC) curves were constructed using MRI-PDFF as the reference standard to assess the diagnostic performance of CAP for discriminating between hepatic steatosis grades. CAP showed significant discriminatory ability for all steatosis grades (all *p* < 0.001). The area under the curve (AUC) values were 0.924 for ≥S1, 0.947 for ≥S2, and 0.837 for S3 steatosis, with corresponding sensitivities of 95.0, 94.9, and 91.7% and specificities of 88.2, 83.8, and 62.5%, respectively. Diagnostic performance was strongest for identifying ≥S1 and ≥S2 steatosis, whereas specificity for S3 was comparatively lower.

**Figure 4:**
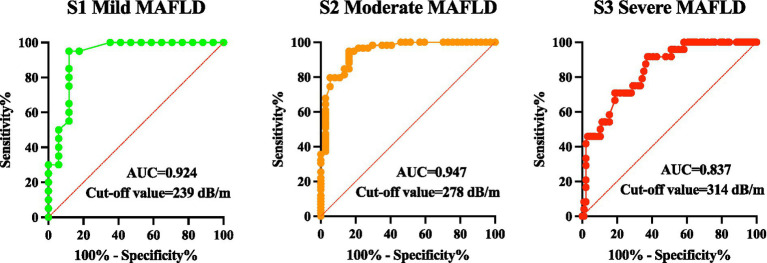
Receiver operating characteristic (ROC) curves of CAP for identifying ≥S1, ≥S2, and S3 steatosis using MRI-PDFF as the reference standard.

The optimal cut-off values were established by maximizing Youden’s index. The 95% confidence intervals (CIs) for these cut-offs were estimated via bootstrap resampling with 2000 iterations. The optimal CAP cut-offs were 239 dB/m (95% CI: 235.5–240.0) for ≥S1 (mild), 278 dB/m (95% CI: 273.0–307.5) for ≥S2 (moderate), and 314 dB/m (95% CI: 303.5–357.5) for S3 (severe) steatosis. The complete diagnostic performance data and corresponding ROC curves are presented in Table 3 and Figure 4.

**Table 3:** Diagnostic performance of controlled attenuation parameter (CAP) for different grades of hepatic steatosis.

Steatosis grade	CAP cut-off (dB/m) (95% CI)	AUC (95% CI)	*p*-value	Sensitivity (%) (95% CI)	Specificity (%) (95% CI)	Youden’s index
≥S1	239.00 (235.50, 240.00)	0.924 (0.826, 1.000)	<0.001	95.00 (76.39, 99.74)	88.24 (65.66, 97.91)	0.832
≥S2	278.00 (273.00, 307.50)	0.947 (0.903, 0.992)	<0.001	94.92 (86.08, 98.61)	83.78 (68.86, 92.35)	0.787
S3	314.00 (303.50, 357.50)	0.837 (0.756, 0.918)	<0.001	91.67 (74.15, 98.52)	62.50 (52.51, 71.53)	0.542

*Magnetic resonance imaging-derived proton density fat fraction (MRI-PDFF) was used as the reference standard. Optimal cut-off values were determined by maximizing Youden’s index.

#### 3.3.1 Additional diagnostic analysis of CAP combined with non-invasive metabolic indices

Additional ROC analyses were performed to evaluate whether combining CAP with non-invasive metabolic indices could improve the diagnosis of ≥S1, ≥S2, and S3 steatosis. Compared with CAP alone, the combined models incorporating FLI, VAI, or TyG did not show a statistically significant improvement in AUC according to DeLong’s test across steatosis grades. Although some combined models showed small numerical changes in sensitivity or specificity, these differences did not translate into a statistically significant overall improvement beyond CAP alone.

However, compared with the corresponding serum-based indices alone, the combined models showed significantly improved diagnostic performance across steatosis grades (all *p* < 0.05). These findings suggest that CAP alone already provided strong discriminatory performance in the present cohort, whereas the combined models showed significantly better diagnostic performance than the serum-based indices alone. Detailed diagnostic performance metrics are presented in Supplementary Table 1.

## 4 Discussion

Against the backdrop of increasing global prevalence of obesity and T2DM, the burden of MASLD has risen substantially and has become a major cause of advanced liver disease in many regions (44). In China, MASLD represents a significant disease burden, with a reported adult prevalence of 29.6% over the past two decades (45). MASLD, metabolic syndrome (MetS), and T2DM are closely interconnected and collectively contribute to the progression of both hepatic and extrahepatic complications, including cardiovascular disease, chronic kidney disease, hepatic decompensation, and HCC (46). Consequently, MASLD has emerged as a critical public health challenge requiring urgent intervention in China (47).

Hepatic steatosis is the fundamental pathological hallmark of MASLD, and accurate assessment of steatosis severity is important for risk stratification and clinical management (48). However, the widespread clinical application of liver biopsy remains constrained by its inherent limitations, including invasiveness, poor reproducibility, and sampling variability (49, 50). These limitations highlight the pressing need for reliable, accurate, and widely accessible non-invasive diagnostic alternatives.

As a non-invasive quantitative technique, MRI-PDFF enables comprehensive assessment of hepatic fat content throughout the entire liver during a single breath-hold by quantifying the proton density ratio of water and fat, thereby effectively circumventing sampling bias (51). This methodology offers several advantages, including standardized acquisition protocols, high reproducibility, strong concordance with histologic findings, and relatively low inter-scanner variability. Consequently, MRI-PDFF has increasingly been used as a non-invasive reference standard in clinical research on hepatic steatosis (52). However, despite its high accuracy for detecting and grading steatosis, the high cost and limited availability of MRI-PDFF restrict its use in routine clinical practice (53).

VCTE is a practical non-invasive technique that provides both LSM and CAP in a single examination (33). Importantly, these two parameters have different clinical roles: LSM mainly reflects liver fibrosis, whereas CAP is the VCTE-derived parameter used to assess hepatic steatosis (24). Thus, for fatty liver grading, CAP is the more directly applicable indicator. Previous studies have demonstrated that CAP has good diagnostic performance for detecting hepatic steatosis and acceptable performance for steatosis grading, supporting its value as a practical non-invasive tool in routine clinical assessment of fatty liver disease (54, 55). VCTE offers several practical advantages, including non-invasiveness, procedural simplicity, rapid acquisition, cost-effectiveness, and good reproducibility, making it attractive for clinical use (7, 56). Nevertheless, CAP may be affected by several factors, including BMI, skin-to-liver capsule distance, transaminase levels, and intercostal space limitations. Moreover, the optimal cut-off values for different steatosis grades have not yet been fully standardized (37, 57).

Against this background, the present study employed MRI-PDFF as a non-invasive reference standard to systematically evaluate the diagnostic performance of CAP across MASLD severity grades and to establish population-specific cut-off values while identifying relevant influencing factors.

In the present study, several anthropometric, biochemical, and elastographic parameters differed significantly across steatosis grades, including WC, BW, BMI, PLT, ALT, AST, GGT, TC, TG, LDL-C, CAP, and LSM. These findings are broadly consistent with the known associations between hepatic steatosis, obesity, dyslipidemia, and liver injury (58, 59).

We additionally evaluated insulin resistance using HOMA-IR, calculated from fasting glucose and fasting insulin, but did not observe a significant difference across steatosis grades or a significant correlation with CAP. These findings further suggest that, in the present cohort, the relationship between CAP and hepatic steatosis severity was not clearly reflected by fasting-state insulin resistance estimated by HOMA-IR. However, this negative result should be interpreted cautiously, as HOMA-IR is an indirect surrogate marker and may also be influenced by cohort characteristics, sample size, and metabolic heterogeneity.

Male participants predominated in this cohort, which may limit the generalizability of the findings to female populations. This sex distribution may partly reflect the epidemiological pattern of MASLD in clinical practice (45). Nevertheless, external validation in larger and more sex-balanced cohorts is warranted.

CAP values demonstrated a progressive increase corresponding to the severity of hepatic steatosis (S3 > S2 > S1 > S0). Correlation analysis identified a strong positive association between CAP values and steatosis grades. A multiple linear regression model was developed, adjusting for potential confounders including age, metabolic parameters (BMI, WC, TG, LDL-C), liver enzyme and platelet count (ALT, PLT), and liver stiffness measurement (LSM) as an indicator of fibrosis. Following adjustment, hepatic steatosis grade remained independently associated with CAP values (*p* < 0.001). These findings suggest that the association between CAP and hepatic steatosis burden remains robust after accounting for metabolic and fibrosis-related covariates. Collectively, these results support the clinical utility of CAP as a non-invasive tool for estimating hepatic steatosis severity in relation to MRI-PDFF-defined steatosis grades (34).

This study established the following optimal CAP cut-off values (95% confidence intervals [CIs]) for hepatic steatosis grading: 239 dB/m (95% CI: 235.5–240.0) for mild (S1), 278 dB/m (95% CI: 273.0–307.5) for moderate (S2), and 314 dB/m (95% CI: 303.5–357.5) for severe (S3) steatosis. The corresponding diagnostic sensitivities were 95.0, 94.9, and 91.7%; specificities were 88.2, 83.8, and 62.5%; and AUC values were 0.924, 0.947, and 0.837, respectively. Overall diagnostic performance was strongest for detecting mild and moderate steatosis, whereas the lower specificity observed for S3 suggests comparatively weaker rule-in performance for severe steatosis.

One possible explanation for the lower specificity observed in S3 steatosis is that severe steatosis may coexist with a greater burden of liver injury and fibrosis (34). In our cohort, participants with S3 steatosis had higher LSM values than those in the S0 group, and liver injury-related parameters such as ALT, AST, and GGT were also elevated compared with S0. These findings suggest that coexisting fibrosis burden may have contributed, at least in part, to the reduced specificity for S3. However, because these differences were not consistently observed between S3 and the intermediate steatosis groups, this interpretation should be made with caution. Other factors, including the use of the M probe alone, the lack of routine skin-to-liver capsule distance assessment, limited sample size, and heterogeneity within the severe steatosis group, may also have influenced the diagnostic performance for S3 steatosis.

The bootstrap-derived confidence intervals provide an estimate of the precision of the proposed cut-off values. The relatively narrow interval for mild steatosis suggests greater stability, whereas the wider intervals for moderate and severe steatosis indicate greater uncertainty, possibly related to sample heterogeneity and the limited number of advanced cases. These findings support cautious interpretation of values near the proposed thresholds and highlight the potential value of integrating CAP with other clinical or imaging parameters in borderline cases.

Compared with previous studies, the current analysis identified a higher CAP cut-off for severe steatosis, accompanied by reduced specificity (62.5%) (Table 4). Several factors may account for these discrepancies, reflecting heterogeneity across studies. First, these discrepancies may be partly attributable to ethnic background, variation in liver disease etiology across study populations, and the use of different reference standards. Methodological differences, including study design and probe selection, may also have contributed to the observed variation in CAP cut-off values. For instance, a meta-analysis by Karlas et al. (31), which included multinational cohorts and patients with viral hepatitis, reported lower cut-off values (S1: 248 dB/m (95% CI: 237–261); S2: 268 dB/m (257–284); S3: 280 dB/m (268–294)) than those observed in our Chinese cohort. These observations are consistent with the possibility that ethnic background and concomitant liver diseases may influence CAP measurements (34, 60). Second, although the cut-off for severe steatosis reported by An et al. (35) (310.5 dB/m) was similar to ours, differences existed for mild and moderate grades (277 dB/m and 290.5 dB/m, respectively). This discrepancy underscores the impact of measurement systems, specifically comparing the iLiv Touch FT1000 device used in their study with the FibroScan® 502 Touch employed herein. Inter-device variability may contribute to such differences (61). Furthermore, obesity prevalence and probe selection criteria may contribute to the elevated cut-off and reduced specificity for S3 steatosis observed in our cohort. As summarized in Table 4, differences in probe selection, study population, and reference standards may all have contributed to the variability in reported CAP cut-off values across studies.

**Table 4:** Comparison of CAP cut-off values for hepatic steatosis grading across different validation studies.

Study	Country/region	Study Population, *n*	Age, years	BMI, kg/m^**2**^	Reference Standard	Device	Probe type	Steatosis grade	CAP cut-off (dB/m)	Sensitivity, %	Specificity, %	AUC
An et al. (35)	China	197	38 ± 8.5	28.8 ± 4.3	MRI-PDFF	iLiv Touch FT1000	M	S1	277	91	92	0.93
								S2	290.5	87	74	0.86
								S3	310.5	72	63	0.73
Karlas et al. (31)	Multiple Countries	2,735	45.4 ± 13.5	25.0 ± 3.9	Liver biopsy	FibroScan	M	S1	248	69	82	0.82
								S2	268	78	81	0.87
								S3	280	88	78	0.88
Eddowes et al. (24)	United Kingdom	380	54 ± 18	33.8 ± 9.2	Liver biopsy	FibroScan 502 Touch	M or XL	S1	302	80	83	0.87
								S2	331	70	76	0.77
								S3	337	72	63	0.70
Chan et al. (30)	Malaysia	79*	*See footnote	*See footnote	Liver biopsy	FibroScan 502 Touch	M	S1	266	91	87	0.94
								S2	273	84	91	0.80
								S3	292	87	50	0.69
							XL	S1	271	95	91	0.97
								S2	276	93	61	0.81
								S3	304	80	56	0.67
Siddiqui et al. (33)	United States	393	51 ± 11	34 ± 6	Liver biopsy	FibroScan 502 Touch	M or XL	S1	285	90	35	0.76
								S2	311	77	57	0.70
								S3	306	80	40	0.58

*Chan et al. (30) study included healthy controls (*n* = 22; age 20.2 ± 1.3 years; BMI 20.5 ± 2.4 kg/m^2^) and NAFLD patients (*n* = 57; age 50.1 ± 10.4 years; BMI 30.2 ± 5.0 kg/m^2^). BMI, body mass index; CAP, controlled attenuation parameter; MRI-PDFF, magnetic resonance imaging-derived proton density fat fraction.

To further explore whether non-invasive assessment could be optimized, we additionally evaluated the performance of CAP combined with simple metabolic indices, including FLI, VAI, and TyG. These combined models did not significantly improve diagnostic performance compared with CAP alone across steatosis grades, suggesting that CAP itself already provided relatively strong discriminatory ability in the present cohort and left limited room for further improvement. However, compared with the serum-based indices alone, the combined models showed significantly better diagnostic performance, indicating that CAP contributed substantial incremental value when integrated with metabolic markers. This pattern suggests that the main advantage of the combined approach was not to outperform CAP itself, but rather to enhance the diagnostic utility of serum-based non-invasive indices. Nevertheless, this finding should be interpreted in light of the modest sample size and imbalanced group distribution, which may have limited the detection of small but potentially meaningful gains beyond CAP alone.

In clinical practice, the M probe remains the standard tool for transient elastography in the general patient population. However, its utility is substantially limited in patients with obesity (BMI > 30 kg/m^2^), as the increased skin-to-liver distance often results in elevated measurement failure rates (62). The XL probe was specifically designed to address this limitation. In the present study, the M probe was used for all participants and SLCD was not routinely measured. Although standard quality-control criteria were met, this approach may have affected CAP accuracy in participants with obesity and may partly explain the relatively high cut-off and lower specificity observed for S3 steatosis (40, 63). These findings highlight the importance of appropriate probe selection in future studies and support the use of the XL probe or SLCD-guided probe choice when feasible. In addition, the exclusive use of the M probe in the present study may limit the generalizability of our findings to broader populations, particularly individuals with obesity in whom XL probe assessment may be more appropriate.

An imbalance in sample distribution was present between healthy controls and patients with MASLD. This likely reflects the clinical reality that MRI-PDFF is more often performed in patients with suspected steatosis than in general screening populations, partly because of its relatively high cost and limited accessibility. Nevertheless, the relatively small number of healthy controls may have affected the precision of some diagnostic estimates, particularly specificity for S3, and should be considered when interpreting the findings. This imbalance may also limit the extrapolation of the present findings to broader screening or general populations.

This study has several strengths. First, it derived MRI-PDFF-referenced CAP cut-off values in a Chinese MASLD cohort, for which such data remain limited. Second, it used MRI-PDFF as a non-invasive reference standard, which reduced the limitations associated with biopsy-based sampling variability. Third, bootstrap resampling was used to quantify the uncertainty of the proposed thresholds.

Several limitations should be acknowledged. First, the number of healthy controls was relatively small, and the distribution across steatosis grades was imbalanced, which may have affected the precision of some diagnostic estimates and limited extrapolation of the findings to broader screening populations. Second, only the M probe was used and SLCD was not routinely measured, which may have influenced CAP accuracy in participants with obesity and may limit the applicability of the findings to populations in whom XL probe assessment would be more appropriate. Third, this was a single-center retrospective study without external validation and with case–control-like sampling rather than a prospective consecutive-enrollment design. Although such a design is acceptable for an initial evaluation of diagnostic performance, it does not represent the most rigorous framework for diagnostic strategy assessment in real-world clinical practice and may introduce selection bias and spectrum effects. Another important limitation is the lack of standardization of ultrasound attenuation-based cut-off thresholds across different vendors and platforms. Even within FibroScan/CAP studies, reported thresholds have varied according to study population and reference standard (31, 35). In addition, other vendor-specific attenuation techniques, such as FibroTouch, ATI, and UGAP, use different threshold systems and, in some cases, different units, which limits direct cross-platform comparability (20, 64). Therefore, these thresholds are not directly interchangeable across devices, which may reduce comparability among studies and limit generalizability. Further international standardization, cross-platform validation, and prospective multicenter studies are needed to establish more consistent attenuation-based thresholds for hepatic steatosis assessment and to validate the proposed cut-off values.

In conclusion, CAP demonstrated good diagnostic performance for identifying ≥S1 and ≥S2 steatosis in this cohort, although further multicenter prospective studies are needed to validate the proposed thresholds, particularly for severe steatosis.

## Data Availability

The raw data supporting the conclusions of this article will be made available by the authors, without undue reservation.
