# Supplementary material for: Diagnostic performance of controlled attenuation parameter for grading hepatic steatosis in MASLD: an MRI-PDFF-referenced study in a Chinese cohort
Source: Front Med (Lausanne). 2026 May 19;13:1836752. doi: 10.3389/fmed.2026.1836752 (PMC13226613; doi:10.3389/fmed.2026.1836752)
Supplement: Supplementary file 1 [file Table_1.DOCX]

**Supplementary Table 1**. Diagnostic performance of CAP, non-invasive metabolic indices, and combined models for identifying ≥S1, ≥S2, and S3 steatosis

| Steatosis grade | Model | AUC (95% CI) | Sensitivity (%) | Specificity (%) | Youden index | DeLong *P* value vs CAP |
| --- | --- | --- | --- | --- | --- | --- |
| ≥S1 | FLI | 0.819  (0.682, 0.956) | 80.00 | 76.50 | 0.565 | 0.189 |
|  | VAI | 0.528  (0.337, 0.719) | 100.00 | 17.60 | 0.176 | 0.001 |
|  | TyG | 0.543  (0.352, 0.733) | 45.00 | 70.60 | 0.156 | 0.001 |
|  | CAP | 0.924  (0.826, 1.000) | 95.00 | 88.24 | 0.832 | Reference |
|  | CAP + FLI | 0.947  (0.877, 1.000) | 95.00 | 88.20 | 0.832 | 0.340 |
|  | CAP + VAI | 0.924  (0.833, 1.000) | 90.00 | 88.20 | 0.782 | 1.000 |
|  | CAP + TyG | 0.926  (0.840, 1.000) | 100.00 | 76.50 | 0.765 | 0.890 |
| ≥S2 | FLI | 0.794  (0.705, 0.884) | 57.60 | 94.60 | 0.522 | 0.001 |
|  | VAI | 0.733  (0.630, 0.836) | 89.90 | 51.40 | 0.412 | <0.001 |
|  | TyG | 0.735  (0.633, 0.838) | 79.70 | 62.20 | 0.419 | <0.001 |
|  | CAP | 0.947  (0.903, 0.992) | 94.92 | 83.78 | 0.787 | Reference |
|  | CAP + FLI | 0.951  (0.911, 0.992) | 94.90 | 83.80 | 0.787 | 0.564 |
|  | CAP + VAI | 0.966  (0.936, 0.997) | 93.20 | 89.20 | 0.824 | 0.205 |
|  | CAP + TyG | 0.960  (0.927, 0.992) | 96.60 | 78.40 | 0.750 | 0.407 |
| S3 | FLI | 0.617  (0.491, 0.743) | 33.30 | 87.50 | 0.208 | <0.001 |
|  | VAI | 0.572  (0.447, 0.698) | 37.50 | 81.20 | 0.187 | <0.001 |
|  | TyG | 0.489  (0.359, 0.619) | 87.50 | 22.90 | 0.104 | <0.001 |
|  | CAP | 0.837  (0.756, 0.918) | 91.67 | 62.50 | 0.542 | Reference |
|  | CAP + FLI | 0.836  (0.754, 0.917) | 91.70 | 62.50 | 0.542 | 0.378 |
|  | CAP + VAI | 0.839  (0.758, 0.920) | 87.50 | 64.60 | 0.521 | 0.813 |
|  | CAP + TyG | 0.843  (0.766, 0.921) | 87.50 | 68.70 | 0.562 | 0.556 |

*Combined models were constructed using binary logistic regression. DeLong *P* values indicate comparisons of AUCs between CAP alone, the individual metabolic indices, and the corresponding combined models within the same steatosis grade. Additional pairwise comparisons between each combined model and the corresponding serum-based index were also performed and showed statistically significant improvement for all combined models (all *P* < 0.05).

*Abbreviations: CAP, controlled attenuation parameter; FLI, fatty liver index; VAI, visceral adiposity index; TyG, triglyceride-glucose index; AUC, area under the receiver operating characteristic curve; CI, confidence interval.*
